# Autophagy activation within inflammatory microenvironment improved the therapeutic effect of MSC-Derived extracellular Vesicle in SLE

**DOI:** 10.1016/j.jare.2025.01.044

**Published:** 2025-01-27

**Authors:** Shuzhen Liao, Fengbiao Guo, Zengzhi Xiao, Haiyan Xiao, Quan-ren Pan, Yugan Guo, Jiaxuan Chen, Xi Wang, Shuting Wang, Haimin Huang, Lawei Yang, Hua-feng Liu, Qingjun Pan

**Affiliations:** a Guangdong Provincial Key Laboratory of Autophagy and Major Chronic Non-communicable Diseases, Clinical Research and Experimental Center, Department of Nephrology, Affiliated Hospital of Guangdong Medical University, Zhanjiang 524000, China; b Department of Clinical Laboratory, State Key Laboratory of Respiratory Disease, The First Affiliated Hospital of Guangzhou Medical University. Guangzhou 510120, China; c Yue Bei People’s Hospital Postdoctoral Innovation Practice Base, Southern Medical University, Guangzhou 510515, China; d Department of Cellular Biology and Anatomy, Medical College of Georgia, Augusta University, Augusta, GA 30912, USA; e James and Jean Culver Vision Discovery Institute, Medical College of Georgia, Augusta University, Augusta, GA 30912, USA; f Department of Radiation Oncology, Yuebei People’s Hospital Affiliated to Shantou University School of Medicine, Shaoguan 512000, China

**Keywords:** Autophagy, Systemic lupus erythematosus, Mesenchymal stem cell, Extracellular vesicles, Rapamycin

## Abstract

**Introduction:**

Developing strategies to improve the therapeutic efficacy of mesenchymal stem cell (MSC)-derived extracellular vesicles (EVs) in autoimmune diseases have garnered increased attention.

**Objectives:**

To evaluate whether rapamycin-induced autophagy within the systemic lupus erythematosus (SLE) inflammatory microenvironment (Rapa-SLE) augments the therapeutic effects of MSC-derived EVs in SLE.

**Methods:**

The therapeutic potential of the resulting EVs (Rapa-SLE-EV) was assessed in MRL/lpr mice. Rapa-SLE-EVs were compared with EVs derived from MSCs from MSCs cultured with EV-depleted fetal bovine serum (FBS-EV), EVs from MSCs cultured with rapamycin-treated FBS (Rapa-FBS-EV), and EVs exposed to SLE serum without rapamycin (SLE-EV). The autoimmune response, renal function, and pathological damage were assessed among the mouse groups. Additionally, mechanistic investigations into the role of the anti-inflammatory protein IDO1 within the EVs.

**Results:**

Interaction with the SLE inflammatory microenvironment triggered autophagy in MSCs, which was further enhanced by rapamycin treatment. Rapa-SLE-EV administration significantly ameliorated the autoimmune response and renal damage in MRL/lpr mice, outperforming other MSC-EV groups. This treatment mitigated key manifestations of SLE, including reduced autoantibody levels, as well as splenomegaly, and lymphadenopathy. Furthermore, Rapa-SLE-EV demonstrated superior suppression of plasma inflammatory cytokines, preserved renal function, mitigated pathological damage, and reduced glomerular immune complex deposition. Mechanistically, Rapa-SLE-EV exhibits exceptional inhibitory effects on SLE-B cell function, benefited by the high expression of the anti-inflammatory protein IDO1, which was confirmed to enter SLE-B cells through EVs.

**Conclusions:**

We developed a novel strategy to improve the therapeutic efficacy of MSC-EVs in SLE and confirmed that the immunomodulatory function of the MSC-EVs is enhanced through autophagic activation and interaction with the SLE serum microenvironment, a process likely benefited by the high expression of IDO1.

## Highlights

• SLE serum triggers the autophagic response of MSCs, while rapamycin regulates the autophagic response of MSCs induced by SLE serum.
• Rapa-SLE-EV is produced by culturing MSCs with SLE serum to simulate the SLE microenvironment and activating autophagy with rapamycin.
• Rapa-SLE-EV showed enhanced therapeutic potential in treating lupus mice and inhibiting B cell function.
• Rapa-SLE-EV enrich more anti-inflammatory protein IDO1, and is a promising therapeutic strategy for SLE.

## Graphical abstract

**Figure:**
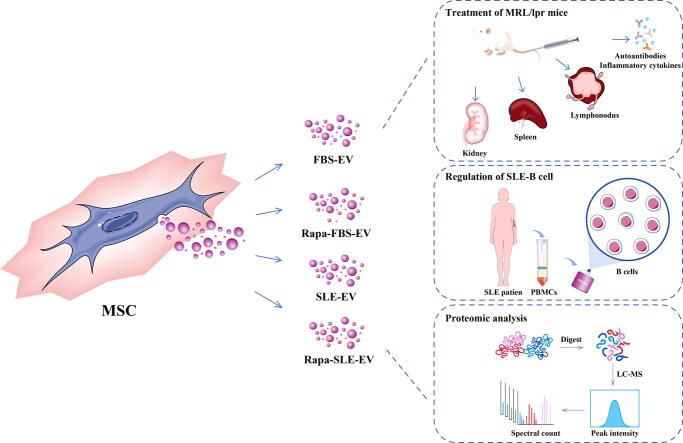

## Introduction

Systemic lupus erythematosus (SLE) is a serious multisystem autoimmune disease that can affect nearly every body system and is considered a classic example of autoimmunity [1]. The efficacy of mesenchymal stem cells (MSCs) transplantation for SLE treatment has been confirmed in numerous studies [2], [3], [4], [5]. The immunomodulatory benefits of MSC transplantation are attributed to the paracrine effects mediated by MSC-derived extracellular vesicles (MSC-EVs) [6], [7], [8].

MSC-EVs can deliver specific molecules to target tissues or organs to induce immune regulatory effects similar to those of MSCs [9]. Clinically, intravenous injection of human placental MSC-EVs significantly improves skin stiffness and dryness in patients with cutaneous chronic graft-versus-host disease [10]. A 2020 clinical trial (CTR2000030261) demonstrated the safety and efficacy of nebulized MSC-EVs in seven COVID-19 pneumonia patients; absorption of pulmonary lesions and shortened hospitalization times were reported with no allergic reactions [11]. Similarly, a pilot study (NCT04276987) evaluating aerosolized allogeneic adipose tissue MSC-EVs in seven severe COVID-19 patients reported good tolerance, no severe adverse events, and pulmonary improvement, with a slight increase in lymphocyte counts observed in six patients [12]. Additionally, a novel MSC-derived product, Exosome-derived Multiple Allogeneic Protein Paracrine Signaling (Exo-d-MAPPS), improved lung function and quality of life in 30 COPD patients with good tolerability, highlighting its potential as a therapy for chronic inflammatory lung diseases [13]. Other ongoing clinical trials investigate EV-based therapies for inflammatory diseases, such as acute respiratory distress syndrome, Crohn’s disease, ulcerative colitis, and periodontitis. Notably, to date, there have been no reported clinical trials specifically investigating the use of MSC-EVs for the treatment of SLE. This gap highlights the critical need for further research to explore the therapeutic potential of MSC-EVs in SLE. MSC-EVs may represent a potential strategy to slow SLE progression and improve lupus nephritis (LN), but their application in these conditions requires further confirmation.

Autophagy is a cellular process responsible for degrading proteins and organelles and is, thus crucial in maintaining cellular homeostasis and promoting survival under stressful conditions [14]. Autophagy can also enhance the immunosuppressive function of MSCs and their anti-inflammatory effects [15]. Specifically, regulating autophagy may impact the secretory ability of MSCs, affecting their function [16], [17]. Meanwhile, inhibiting autophagy weakens the therapeutic effect of EVs secreted by adipose-derived stem cells on lipopolysaccharide-induced pulmonary microvascular endothelial cell barrier damage and acute lung injury [18]. However, whether autophagy mediates the therapeutic effects of MSC-EVs in SLE remains unknown.

Different microenvironments have unique effects on the proliferation, differentiation, metabolism, and functional activities of MSCs [19]. The immunosuppressive function of MSCs requires inflammation [20]. Cytokine priming is a useful strategy for harvesting anti-inflammatory MSC-EV for clinical applications [21], [22], [23]. The complex microenvironment influences the paracrine effect of MSC transplantation in SLE, and various factors should be comprehensively considered. Therefore, this study simulated the effect of the human SLE serum microenvironment on MSCs and activated autophagy to examine whether it enhances the immunosuppressive function of MSC-EV. We aimed to determine whether autophagy activation within the inflammatory microenvironment can augment the therapeutic effects of MSC-EVs in SLE.

## Materials and methods

### Patients and clinical parameters

Based on the modified SLE classification criteria formulated by the American College of Rheumatology in 1997, 46 patients with SLE were enrolled in this study at the Department of Nephrology and Rheumatology between December 2021 and July 2022. SLE was assessed according to the SLE disease activity index (SLEDAI) 2000 (SLEDAI score of > 6). The serum EVs from newly diagnosed SLE patients were removed by overnight ultracentrifugation at 120,000 × *g* and 4°C for MSCs culture. Table 1 summarizes the patients' age, sex, positivity rate for immunological parameters, SLEDAI score, and treatment undergone. This study was approved by the institutional ethics committee (Approval no. YJYS2021069). Written informed consent was obtained from all the patients.

**Table 1:** Demographic characteristics of patients with SLE.

Demographic variable	SLE patients (n = 46)
Age	
Range, years	18–60
Mean, SD	32.95 ± 11.95
Sex	
F/M (%)	42 (91 %)/4 (9 %)
Immunological parameters	
Anti-dsDNA positive (%)	34 (73.9 %)
Anti-nuclear positive (%)	44 (95.6 %)
C3 Mean, SD	0.58 ± 0.22 (g/L)
C4 Mean, SD	0.14 ± 0.10 (g/L)
SLEDAI score	
Mean, SD	13.51 ± 5.78
Median (Min, Max)	12 (7, 28)
Patients	
Newly diagnosed	10 (21.7 %)
Glucocorticoids	3 (6.5 %)
Hydroxychloroquine	36 (78.2 %)
Azathioprine	34 (73.9 %)
Cyclophosphamide	7 (15.2 %)
Methotrexate	11 (23.9 %)
Mycophenolate Mofetil	9 (19.5 %)
Belimumab	4 (8.7 %)

This study was approved by our hospital's ethics committee. Written informed consent was obtained from all the patients.

### Human umbilical cord MSC (hUC-MSC) culture and treatment

hUC-MSCs (generation 5–7) were obtained from Hunan Yuanpin Biotech Co. Ltd. (Changsha, China) and cultured in minimum essential medium (MEM) alpha basic (Gibco, Grand Island, NY, USA) at 5 % CO_2_ and 37 °C. The physiological and disease states were simulated by adding 10 % EV-depleted FBS (Vivacell, Israel) or SLE serum (SLEDAI score > 6) respectively. Rapamycin (5 μM; Sigma-Aldrich, St. Louis, MO, USA) induced autophagy activation.

Western blotting was performed to assess the levels of autophagy-related proteins, namely phosphorylated mammalian target of rapamycin (p-mTOR: Rabbit anti-human, CST, USA), LC3B (rabbit anti-human; Sigma-Aldrich), and P62 (mouse anti-human; Abcam, Cambridge, UK).

### Isolation and identification of MSC-EVs

Supernatants from each group were collected and centrifuged (4 °C) at 300 × *g* for 10 min, 2000 × *g* for 10 min, and 10,000 × *g* for 30 min. EVs were then pelleted at 100,000 × *g* for 1 h, washed once in phosphate-buffered saline (PBS), and passed through a 0.22 μm filter. In all experiments, the EVs were used immediately after ultracentrifugation (Optima XE-100-IVD; Beckman Coulter, Danvers, MA, USA).

The ultrastructure and shape were confirmed using transmission electron microscopy (TEM). Western blotting was performed using antibodies specific for CD9 (anti-CD9 antibody; Abcam), CD63 (anti-CD63 antibody; Abcam), TSG101 (anti-TSG101 antibody; Abcam), and calnexin (anti-calnexin antibody; Abcam) to confirm the identity of EVs (CD9^+^CD63^+^TSG101^+^Calnexin^–^). The nanoparticle tracking analysis (NTA) software (Nanosight NS300, Malvern, UK) was used to detect the size distribution and concentration of the EVs. The rapamycin concentration in Rapa-FBS-EVs was determined using high-performance liquid chromatography (HPLC). The MSC-EVs were divided into four groups: FBS-EVs derived from MSCs cultured in FBS without rapamycin; Rapa-FBS-EVs derived from derived from MSCs cultured in FBS with rapamycin; SLE-EVs derived from derived from MSCs cultured in SLE serum without rapamycin; and Rapa-SLE-EVs derived from derived from MSCs cultured in SLE serum with rapamycin.

### Preparation and cultivation of cells

Peripheral blood mononuclear cells (PBMCs) were isolated from patients with active SLE using Lymphoprep (STEMCELL Technologies, Vancouver, Canada) according to the manufacturer's protocol. Peripheral blood CD19-expressing B cells were selected via negative selection using the EasySep Human B-Cell Isolation Kit (STEMCELL Technologies, Canada), according to the manufacturer's protocol (final purity: > 95 %). The cells were cultured in 5 % CO_2_ at 37 °C, with complete X-Vivo 15 medium supplemented with 10 % FBS and 1 % penicillin/streptomycin.

### Western blotting

To detect the autophagy-related proteins in MSCs and marker proteins of MSC-EVs, spleen tissue was ground and lysed. Cell lysate protein samples were boiled at 95 °C for 10 min, resolved using sodium lauryl sulfate–polyacrylamide gel electrophoresis, and then transferred onto a polyvinylidene fluoride membrane. The membranes were blocked with 5 % skim milk, incubated with primary antibodies overnight at 4 °C, washed, and then incubated with horseradish peroxidase-conjugated secondary antibody. The chemiluminescence reaction with luminol was observed using an Azure C500 Western Blot Imaging System and analyzed by ImageJ.

### Animal studies

Six MRL/MpJ and 30 MRL/lpr mice (female, aged 3–4 weeks) were purchased from Changzhou Cavens Model Animal Co., Ltd. [license no. SCXK (Su) 2021–0013]. All animal experiments were approved by the institutional Laboratory Animal Ethical Committee (LAEC; Approval no. GDY2103031). Mice were housed in a specific pathogen-free facility at 22–25 °C with a 12-h light/dark cycle, 40–60 % humidity, and free access to water and feed. The mice were divided into six groups: (1) MRL/MpJ (n = 6), (2) PBS (n = 6), (3) FBS-EV (n = 6), (4) Rapa-FBS-EV (n = 6), (5) SLE-EV (n = 6), and (6) Rapa-SLE-EV (n = 6). MSC-EVs were administered via the tail vein on weeks 12 and 14 (100  μg in 100  μL PBS per mouse). The mice were euthanized through intraperitoneal injection of pentobarbital sodium (100 mg/kg), and cervical dislocation after they failed to respond to compression. Tissue and blood samples were then collected.

### In vivo tracing of MSC-EVs

MSC-EVs were labeled with a PKH26 fluorescent dye (PKH26 Fluorescent Cell Linker Kit, Sigma-Aldrich). In week 18, MRL/lpr mice in each group were injected with the appropriate MSC-EVs via the tail vein. After 24 h, they were euthanized (n = 3), and the kidneys and spleens were collected, flash-frozen, and sectioned. After staining with 4′,6-diamidino-2-phenylindole (Sigma-Aldrich), the slices were observed under a confocal microscope (FV 3000, Olympus, Japan).

### Enzyme-linked immunosorbent assay (ELISA)

Serum levels of anti-dsDNA IgG and anti-nuclear antibodies (ANA) were measured using a mouse anti-dsDNA antibodies total IgG ELISA Kit (Catalog No. 5110, Alpha Diagnostic, San Antonio, TX, USA) and a mouse ANA Total Ig ELISA Kit (Catalog No. 5210, Alpha Diagnostic, San Antonio, Texas, USA), respectively, according to the manufacturer's instructions.

### Spleen/body weight measurement

The total body weight of 18-week-old MRL/lpr mice was measured before euthanasia. The spleen was collected after euthanasia and weighed and dissected.

### Flow cytometry (FCM)

Peripheral blood from mice was collected, and red blood cells were lysed. The samples of cells was co-stained with APC-Cy7 Rat Anti-Mouse CD19 (Catalog No. 557655, BD Biosciences) and PerCP/Cyanine5.5 anti-mouse CD138 (Catalog No. 142530, BioLegend), APC anti-mouse IgD (Catalog No. 405714, BioLegend), and FITC anti-mouse IgG (Catalog No. 406001, BioLegend) antibodies for 30  min at 4 °C protected from light to detect the plasma cells proportion. The signals were acquired on a FACS Celesta Flow Cytometer (BD Biosciences). The acquired data were analyzed and visualized using FlowJo v10.4.

### Determination of plasma cytokine levels

Plasma levels of inflammatory cytokines, including interferon-gamma (IFN-γ), interleukin (IL)-1β, IL-2, IL-4, IL-5, IL-6, IL-10, IL-12 (p70), IL-13, IL-17A, chemokine (C-X-C motif) ligand (CXCL)1, chemokine ligand (CCL)2, CXCL2, B-cell activating factor (BAFF), and granulocyte–macrophage colony-stimulating factor (GM-CSF) were assayed using a mMag Luminex Assay (R&D Systems, Minneapolis, MN, USA), according to the manufacturer's recommendations.

### Renal function

The mice were housed overnight (12-h) in metabolic cages to collect urine samples. The urine protein levels were assessed using an automatic analyzer (Cobas 8000, Roche, Switzerland). Serum creatinine and urea nitrogen were measured using creatinine assay kits (No: C011-2–1 and No: C013-2–1, respectively; Nanjing Jiancheng Bioengineering Institute; Nanjing, China).

### Pathological assessment of kidneys

Kidneys were fixed in 4 % paraformaldehyde (PFA) in PBS, dehydrated, and embedded in paraffin. After dewaxing, 3 μm sections were stained with hematoxylin and eosin (H&E), periodic acid-Schiff (PAS), and Masson's trichrome stain consecutively. The scores assigned to mesangial proliferation and PAS deposition ranged from 0 to 4, indicated severity (0, absent; 1, mild; 2, mild-moderate; 3, moderate;and 4, severe). The grades of crescents, tubular lesions (atrophy, casts, dilatation, and inflammatory infiltrates), and vasculitis were each scored on a scale of 0–4 scale, representing the extent of their presence in the kidney sections.

### Immunofluorescence

Rapid-frozen sections of mouse kidney or spleen tissue were washed with PBS and fixed with 4 % PFA. After washing again with PBS, 5 % bovine serum albumin was used to block non-specific antigens. The primary and fluorescent secondary antibodies were incubated at 4 °C overnight and at 20 °C for 1 h, respectively. The primary antibodies used were goat anti-mouse IgG, Alexa Fluor 647 (A21202; Invitrogen, Carlsbad, CA, USA), IgM Antibody (NBP2-62012; Novus Biologicals, Littleton, CO, USA), fluorescein isothiocyanate-conjugated complement component C3 (NB200-540 AF488; Novus), anti-CD19 antibody (ab245235; Abcam) and anti-Indoleamine 2, 3-dioxygenase antibody (ab311847; Abcam). Nuclei were stained with diluted 4′,6-diamidino-2-phenylindole solution for 10 min and sealed. Images were acquired using a laser-scanning confocal microscope (FV 3000, Olympus, Tokyo, Japan).

### TEM

Murine kidney tissues (1 mm × 1 mm) were incubated in 2 % glutaraldehyde and 4 % formaldehyde in 0.1 mL PBS (PH 7.2) overnight at 4 °C and further fixed in 1 % osmium tetroxide in s-collidine buffer. Sections (0.1  μm) were stained with lead citrate and uranyl acetate, and examined at 80  kV using a transmission electron microscope (JEM-1400, MA JEOL Ltd, Japan).

### In vitro uptake of Rapa-SLE-EVs

PKH26-labeled Rapa-SLE-EVs (50 μg/mL) were co-cultured with PBMCs from SLE patients for 22 and 46 h. Cells were stained with directly conjugated CD3 and CD19 antibodies for 2 h, and 4′,6-diamidino-2-phenylindole for 10 min. They were then washed, and resuspended. Fluorescence signals were observed under a confocal microscope.

### B cells differentiation, activation, proliferation, and antibody secretion

To assess B cell differentiation, proliferation, and activation, PBMCs from SLE patients were co-cultured with MSC-EV (50 μg/mL) for 96 h, 72 h, and 48 h, respectively. After incubation, cell suspensions were collected, centrifuged, and the supernatants were stored at −80 °C. The cells were stained with antibodies at 4 °C in the dark for 30 min, and fluorescence signals were analyzed via flow cytometry. FITC mouse anti-human CD19, PE mouse anti-human CD27, APC mouse anti-human CD38, PerCP-Cy5.5 mouse anti-human IgD, PE-Cy7 mouse anti-human CD80, APC mouse anti-human CD86, PE-Cy7 mouse anti-human CD69, and Alexa Fluor 647 mouse anti-Ki-67 were purchased from BD Biosciences. IgG levels in the supernatants were measured according to the manufacturer’s instructions (RayBiotech, USA).

### Label-free protein mass spectrometry and analysis

Culture supernatants were collected from different treatment groups, and MSC-EVs were isolated for label-free protein mass spectrometry and analysis. After quality control of protein samples, they were analyzed using a mass spectrometer. Proteome Discoverer 2.2 (Thermo-Fisher Scientific, Waltham, MA, USA) was used to identify peptide signals in the LC-MS data, followed by database retrieval on MS2 of all peptide signals to obtain accurate secondary sequence confirmation and achieve quantitative analysis through signal strength. The limma R package was used to analyze the differences. For differential expression analysis between two samples, we used the significance A/B method to calculate the significance of the differences. Using | log2 (FC) | ≥ 0.5849625; *P* ≤ 0.01 was used as the screening criterion. Cluster analysis of differential protein expression levels was performed using R software (version 4.2) pheatmap package (version 1.0.12). Based on the Kyoto Encyclopedia of Genes and Genomes (KEGG) database, differential proteins were analyzed for pathway enrichment using R software (version 4.2) ggplot2 package (version 3.2.2).

### Recombinant adenovirus infection of MSCs

The indoleamine 2,3-dioxygenase-1 (IDO1)-overexpression (OE) adenovirus, siRNA-IDO1 adenovirus, and corresponding negative controls were purchased from Jikai Gene Technology Co., Ltd. (Shanghai, China). When the cells reached 50 % confluency, half of the culture medium was replaced with fresh medium. An appropriate volume of virus solution and co-staining reagent was added according to the determined MOI value, gently mixed, and used for infection. After 4 h, the culture volume was restored. At 24 h post-infection, the medium was replaced with fresh complete culture medium, and cultivation was continued. After 24–48 h of infection, the expression efficiency of enhanced green fluorescent protein (EGFP) was observed under a fluorescence microscope. Subsequently, cells were collected to verify the expression of the target gene. The culture supernatant was collected, and EVs were extracted for validation and subsequent functional experiments.

### Statistical analysis

Data were presented as the mean ± standard deviation (SD) of at least three independent experiments. The Kolmogorov–Smirnov and Shapiro–Wilk tests were performed to assess the normality of the data distribution. For comparisons between the two groups, Student's *t*-tests and Welch *t*-tests were used when the variance was uniform and uneven, respectively. For comparisons involving multiple groups, a one-way or two-way analysis of variance (ANOVA) was performed, followed by appropriate post hoc tests (e.g., Tukey's test or Bonferroni correction) to account for multiple comparisons and control the risk of Type I error. Statistical significance was set at *P* < 0.05. Data analysis and graph generation were performed using GraphPad Prism 8.0.2 (GraphPad Software, San Diego, CA, USA).

## Results

### Autophagy activation in MSCs and characterization of MSC-EVs

**Fig. 1:**
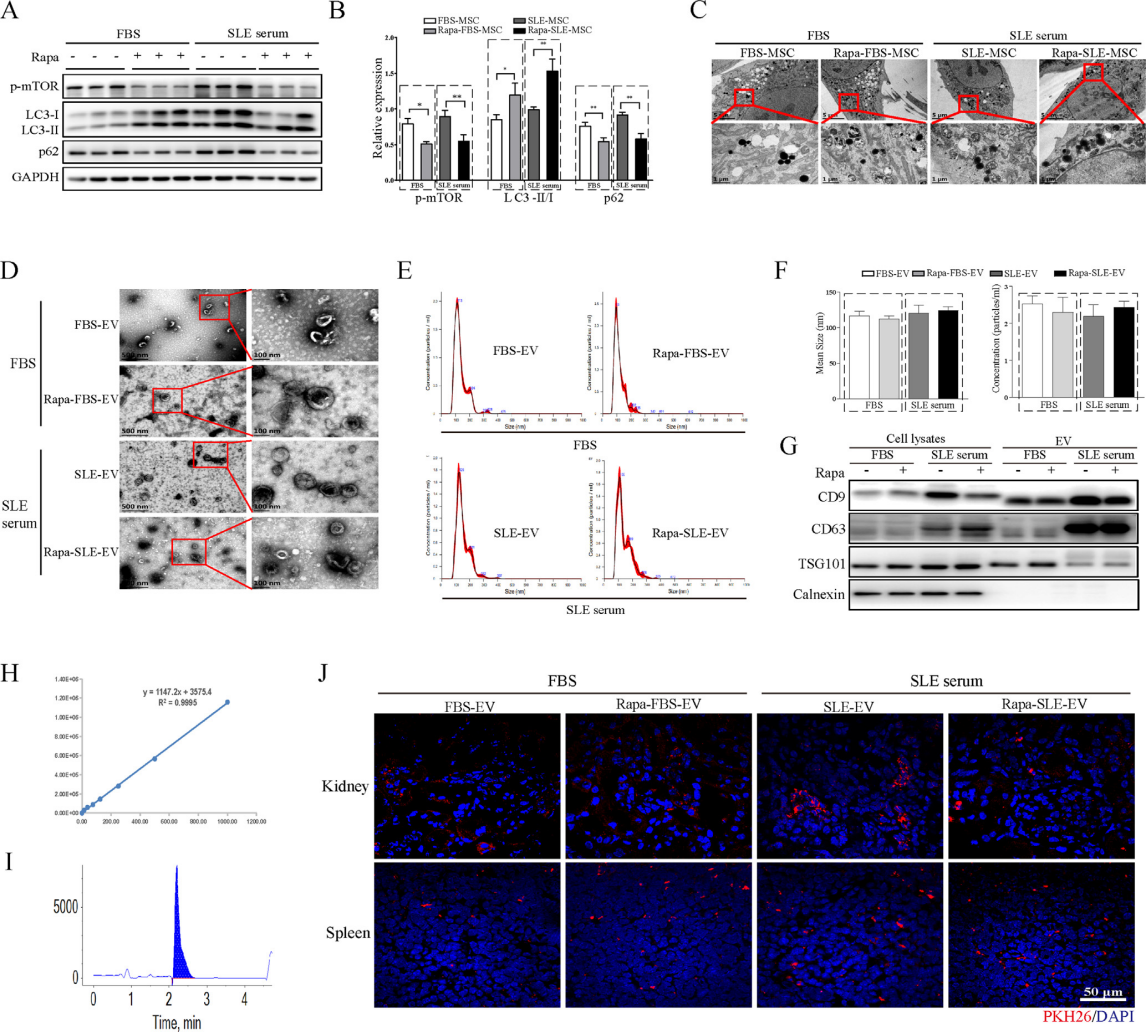
**Activation of autophagy in MSCs and characterization of MSC-EVs.** (A) Representative western blot of p-mTOR, LC3, and p62 in MSCs. (B) Statistical analysis of p-mTOR, LC3, and p62 expression. (C) Representative TEM images of MSC autophagic vacuoles. (D) TEM analysis of vesicles derived from MSCs. (E) NTA of vesicles derived from MSCs. (F) Comparison of mean size and total particle counts in each group of vesicles derived from MSCs. (G) Representative western blot analysis of CD9, CD63, TSG101 and calnexin in MSC-EVs. (H) Linear regression equation of the rapamycin standard curve. (I) Chromatographic peak of rapamycin in MSC-EVs detected using HPLC. (J) Distribution of MSC-EVs labeled with PKH-26 in the kidney and spleen 24 h after tail vein injection. Results are expressed as the mean ± SD of three independent experiments. *P < 0.05, **P < 0.01. FBS-EVs: from MSCs cultured in FBS; Rapa-FBS-EVs: from MSCs cultured with rapamycin and FBS; SLE-EVs: from MSCs cultured with SLE serum; Rapa-SLE-EVs: from MSCs cultured with rapamycin and SLE serum.

Western blotting results revealed that the microtubule-associated protein 1 light chain 3 (LC3)-II level was markedly increased in MSCs cultured with SLE serum compared with the control cells cultured with FBS. Simultaneously, the abundance of the protein Sequestosome 1 (SQSTM1/P62) autophagy receptor also increased, which may be a complex component of SLE serum, obstructing the MSC autophagic flux (Fig. 1A). Meanwhile, rapamycin pretreatment reversed the increase in P62 protein expression levels in MSCs in the SLE serum and further increased the LC3-II/I ratio (Fig. 1B). Under TEM, more autophagic vacuoles were detected in MSCs cultured with SLE serum than in those cultured with FBS (Fig. 1C). In an FBS environment, the abundance of p-mTOR and P62 in MSCs pretreated with rapamycin was also effectively reduced, whereas LC3-II expression was increased. These data indicate that the SLE microenvironment triggered autophagy in MSCs. The benefits of MSC transplantation in treating lupus may be related to autophagy activation. Moreover, rapamycin treatment effectively activated autophagy in MSCs, indicating that rapamycin regulates the SLE serum-induced autophagic response in MSCs.

The isolated MSC-EVs were analyzed per the requirements of “Minimal information for studies of extracellular vesicles 2018 [24].” TEM analysis revealed that the MSC-EVs had a nearly spherical morphology (Fig. 1D) and were between 70 and 150 nm (Fig. 1E). In FBS and SLE serum with or without rapamycin pre-conditioning, MSC-EVs had comparable mean size and total particle counts (Fig. 1F). Western blotting confirmed the enrichment of CD9, CD63, and TSG101 on MSC-EVs. Additionally, the levels of calcium junction proteins expressed in EVs were markedly lower than those in the cell lysate samples. Moreover, the EVs derived form MSCs treated with SLE serum expressed higher levels of CD9 and CD63 and lower levels of TSG101 than those derived from MSCs treated with FBS (Fig. 1G). These results indicate that the four successfully isolated MSC-EVs have different characteristics. Additionnally, autophagy activation and SLE serum treatment may be involved in the packaging and synthesis of MSC-EVs.

To exclude the effect of rapamycin on MRL/lpr mice, the concentration of rapamycin in MSC-EVs was determined using HPLC. The rapamycin standard curve followed a linear regression equation of Y = 1147.2X + 3575.4, with R^2^ = 0.9995 (Fig. 1H). The chromatographic peak for rapamycin in the MSC-EVs was detected after 2 min (Fig. 1I). Statistically, no difference was observed in rapamycin concentrations between Rapa-FBS-EVs and Rapa-SLE-EVs (77.68 ± 0.25 *vs* 79.44 ± 1.27 ng/mL).

SLE is a systemic autoimmune disease. Therefore, MSC-EVs were injected into MRL/lpr mice via the tail vein. To demonstrate the directional effect and absorption of MSC-EVs by lesion tissue, red fluorescence (PKH-26) was observed in the kidney and spleen via confocal microscopy 24 h post-injection (Fig. 1J).

### Autophagy activation enhances the therapeutic effects of MSC-EVs on autoantibodies, splenomegaly, and lymphadenopathy in MRL/lpr mice

**Fig. 2:**
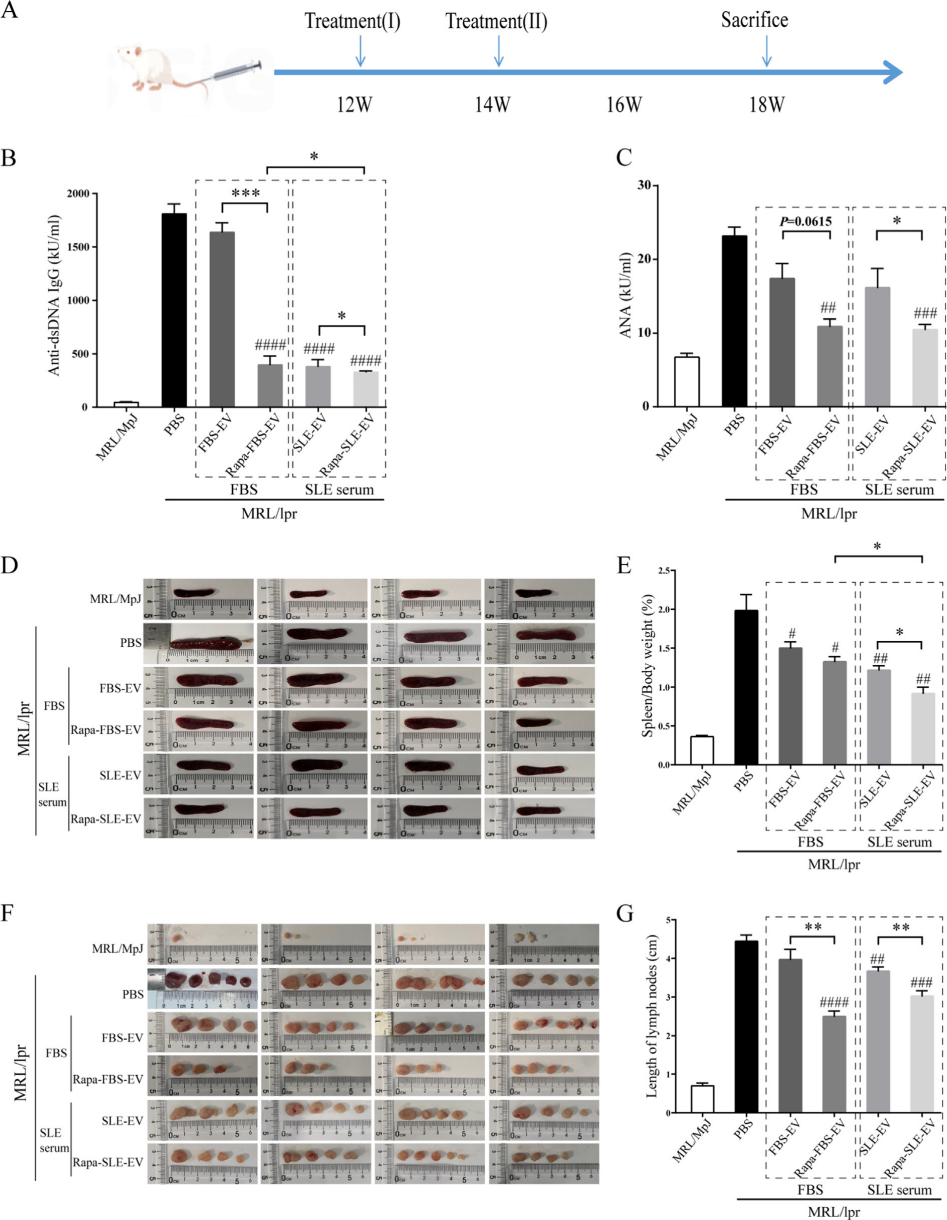
**Autophagy activation enhances the therapeutic effects of MSC-EVs on autoantibodies, lymphadenopathy, and splenomegaly in MRL/lpr mice.** (A) Experimental flowchart of MSC-EV treatment. (B) Concentration of anti-dsDNA IgG antibodies. (C) Concentration of ANA antibodies. (D) Images of spleens. (E) Statistical analysis of spleen/body weight ratio. (F) Images of lymph nodes. (G) Statistical analysis of lymph node diameter. ^#^*P* < 0.05, ^##^*P* < 0.01, ^###^*P* < 0.001, ^####^*P* < 0.0001 *vs* the PBS group. *P < 0.05, **P < 0.01, ***P < 0.001 between groups. FBS-EVs: from MSCs cultured in FBS; Rapa-FBS-EVs: from MSCs cultured with rapamycin and FBS; SLE-EVs: from MSCs cultured with SLE serum; Rapa-SLE-EVs: from MSCs cultured with rapamycin and SLE serum.

The experimental scheme is shown in Fig. 2A. MRL/lpr mice developed massive lymphadenopathy and autoimmunity, including elevated levels of autoantibodies, splenomegaly, and lymphoproliferation. Compared with MRL/MpJ mice, the PBS-treated mice showed an increase in serum anti-dsDNA IgG levels (MRL/MpJ group, 48.4 ± 4.4 kU/mL; PBS group, 1811.47 ± 203.96 kU/mL). The Rapa-FBS-EV MRL/lpr group exhibited significantly reduced serum anti-dsDNA IgG level compared with the EV group without autophagy activation (Rapa-FBS-EV group *vs.* FBS-EV group, *P* < 0.05). Meanwhile, the Rapa-SLE-EV and SLE-EV treatment groups showed significantly reduced serum anti-dsDNA IgG levels in MRL/lpr mice compared with PBS-treated mice; the therapeutic effect of Rapa-SLE-EVs was more significant than that of SLE-EVs (Rapa-SLE-EV group *vs.* SLE-EV group, *P* < 0.05). Notably, mice in the Rapa-SLE-EV group had lower levels of anti-dsDNA IgG than those in the Rapa-FBS-EV group (Rapa-SLE-EV group *vs.* Rapa-FBS-EV group, *P* < 0.05; Fig. 2B). Additionally, compared with the FBS-EV and SLE-EV groups without activated autophagy, the Rapa-FBS-EV and Rapa-SLE-EV groups exhibited lower ANA antibody levels, representing superior therapeutic effects (Rapa-FBS-EV group *vs.* FBS-EV group, *P* = 0.0615; Rapa-SLE-EV group *vs.* SLE-EV group, *P* < 0.05) (Fig. 2C).

At 18 weeks of age, the mice were euthanized, and the spleen weight ratios (Fig. 2D) and mandibular lymph nodes (Fig. 2E) were assessed. PBS-treated mice showed lymphadenopathy and splenomegaly compared to MRL/MpJ mice. MSC-EV treatment inhibited splenomegaly in MRL/lpr mice, and the corresponding spleen/body weight ratio was significantly lower than in PBS-treated mice. The spleen weight ratio in the Rapa-FBS-EV group significantly decreased compared to the EV group; However, that in the Rapa-SLE-EV group decreased more significantly compared to the Rapa-FBS-EV group (Rapa-SLE-EV group *vs.* Rapa-FBS-EV group, *P* < 0.05; Fig. 2E). Similarly, MSC-EV treatment reduced lymph node enlargement; the therapeutic efficacy of Rapa-SLE-EV was superior (Fig. 2F).

These results indicate that autophagy activation enhanced the therapeutic benefits of MSC-EVs on autoantibodies, lymphadenopathy and splenomegaly with SLE. MSC-EV (Rapa-SLE-EV), which further activates autophagy in the serum environment, seems more potent.

### Autophagy activation enhances the inhibitory effect of MSC-EVs on peripheral blood B-cell differentiation

**Fig. 3:**
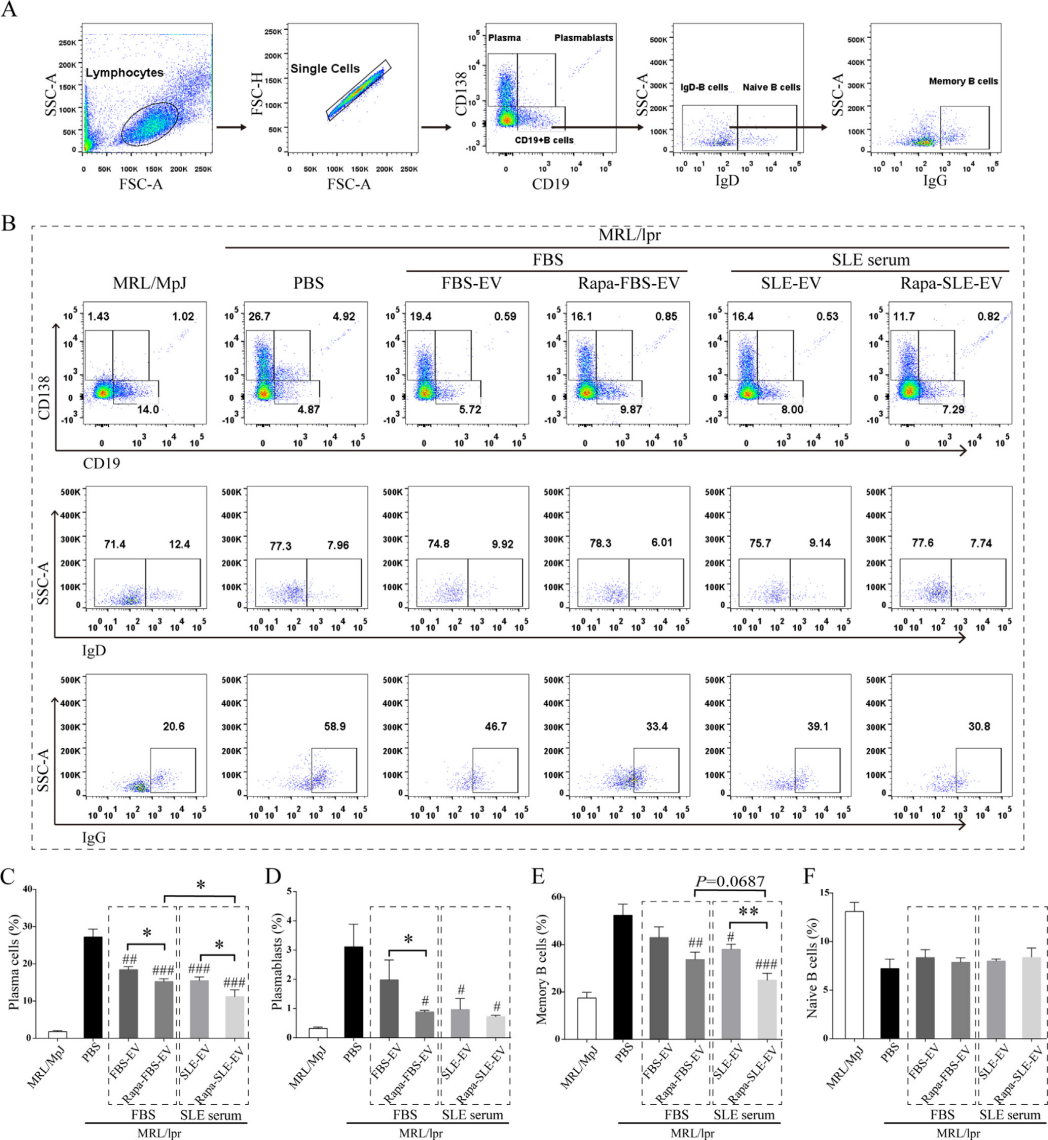
**Autophagy activation enhances the inhibitory effect of MSC-EVs on peripheral blood B-cell differentiation.** (A) Flow cytometry gating strategy. (B) Representative flow cytometry plots showing plasma cells, plasmablasts, naïve B cells, and memory B cells. (C) Statistical analysis of plasma cell proportion; (D) Statistical analysis of plasmablast proportion. (E) Statistical analysis of memory B-cell proportion; (F) Statistical analysis of naïve B-cell proportion. ^#^*P* < 0.05, ^##^*P* < 0.01, ^####^*P* < 0.001 *vs* the PBS group. *P < 0.05 between groups. FBS-EVs: from MSCs cultured in FBS; Rapa-FBS-EVs: from MSCs cultured with rapamycin and FBS; SLE-EVs: from MSCs cultured with SLE serum; Rapa-SLE-EVs: from MSCs cultured with rapamycin and SLE serum.

Based on observed morphological and histopathological changes in the spleen, we hypothesized that autophagy activation influences the inhibitory effect of MSC-EVs on the differentiation of B-cell subpopulations. Plasma cells, plasmablasts, and memory B cells play crucial roles in SLE pathogenesis [25], [26]. The data showed that all MSC-EV treatment groups could inhibit the differentiation of plasma cells, plasmablasts, and memory B cells to varying degrees. Compared to the PBS group, all MSC-EV treatment groups exhibited significantly reduced the proportions of plasma cells (PBS group: 27.25 ± 5.18 %, FBS-EV group: 18.5 ± 1.74 %, Rapa-FBS-EV group: 15.28 ± 1.75 %, SLE-EV group: 15.52 ± 2.21 %, Rapa-SLE-EV group: 11.2 ± 4.38 %). Furthermore, under FBS and SLE serum culture conditions, the proportion of plasma cells was significantly reduced in the Rapa-FBS-EV and Rapa-SLE-EV treatment groups compared with the FBS-EV and SLE-EV treatment groups (Rapa-FBS-EV group *vs.* FBS-EV group, *P* < 0.05; Rapa-SLE-EV group *vs.* SLE-EV group, *P* < 0.05). The proportion of plasma cells was significantly lower in the Rapa-SLE-EV group compared with the Rapa-FBS-EV group (Rapa-SLE-EV group *vs.* Rapa-FBS-EV group, *P* < 0.05; Fig. 3B and C).

Regarding plasmablasts and memory B cells, the inhibitory effects of Rapa-FBS-EVs, SLE-EVs, and Rapa-SLE-EVs were significantly greater than those of FBS-EVs (Fig. 3 B, D, and E). Notably, MSC-EV treatment had no significant effect on the proportion of naïve B cells (Fig. 3 B and F). This suggested a selective inhibitory effect that helped reduce the production of pathological autoantibodies while preserving the normal function of naïve B cells.

These results suggest that autophagy activation enhances the immunomodulatory function of MSC-EVs; hence, activating autophagy in the SLE microenvironment may further optimize the function of MSC-EVs.

### Autophagy activation enhances the inhibitory effect of MSC-EVs on plasma inflammatory cytokines

**Fig. 4:**
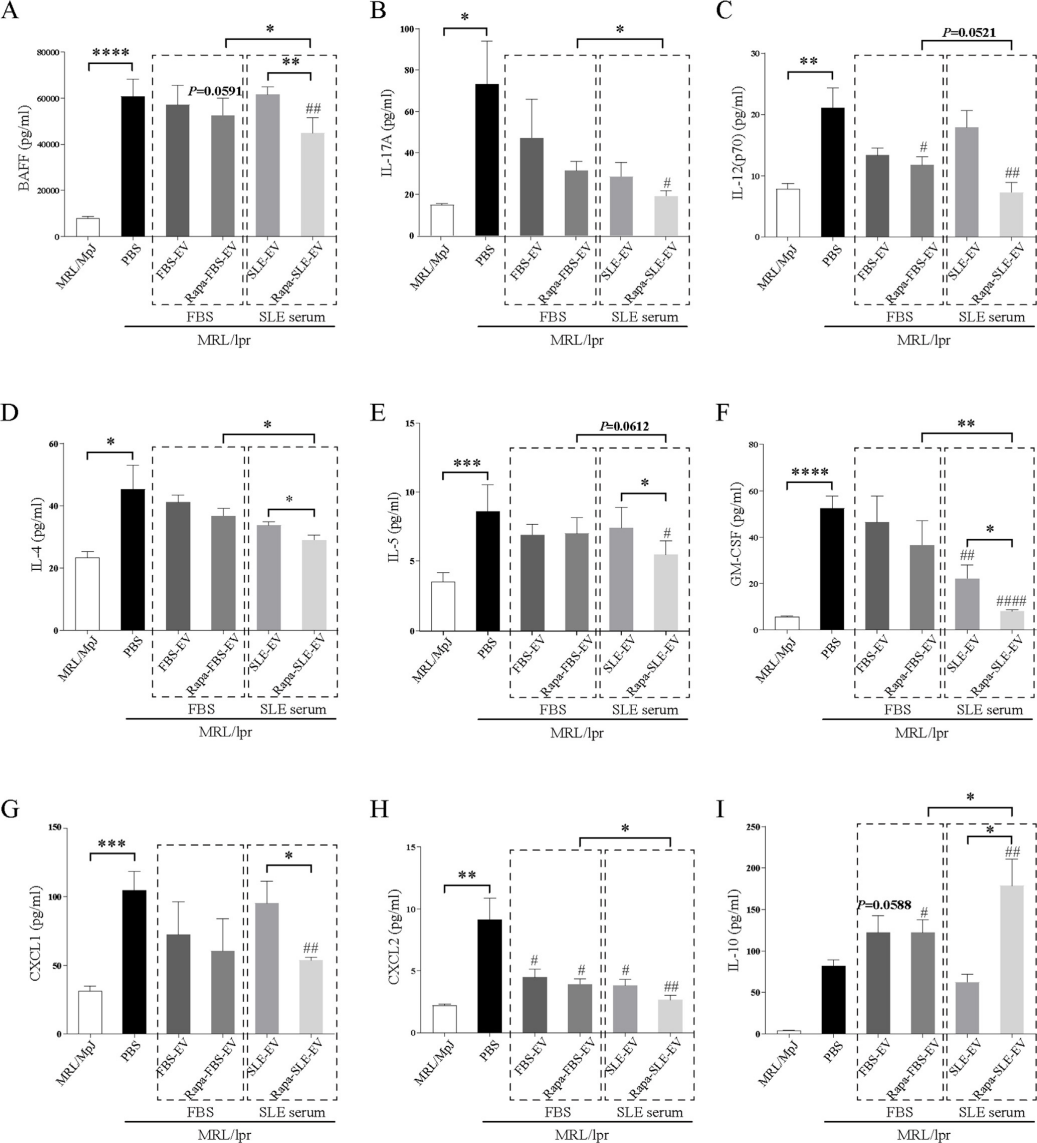
**Autophagy activation enhances the inhibitory effect of MSC-EVs on plasma inflammatory factors.** Statistics of the plasma levels of BAFF (A), IL-17A (B), IL-12p70 (C), IL-4 (D), IL-5 (E), GM-CSF (F), CXCL1 (G), CXCL2 (H), and IL-10 (I). ^#^*P* < 0.05, ^##^*P* < 0.01, ^####^*P* < 0.0001 *vs* the PBS group. *P < 0.05, **P < 0.01, ***P < 0.001, ****P < 0.0001 between groups. FBS-EVs: from MSCs cultured in FBS; Rapa-FBS-EVs: from MSCs cultured with rapamycin and FBS; SLE-EVs: from MSCs cultured with SLE serum; Rapa-SLE-EVs: from MSCs cultured with rapamycin and SLE serum.

To characterize the effect of MSC-EV inhibition in MRL/lpr mice, we analyzed the differential expression of plasma cytokines in week 18. No significant differences were observed between the MSC-EV- and PBS-treated groups in IL-2, IL-13, IL-1β, and IFN-γ levels (data not shown). However, varying degrees of reduction were observed in the plasma levels of BAFF, IL-17A, IL-12p70, IL-4, IL-5, GM-CSF, CXCL1, and CXCL2 (Fig. 4A–H) in the MSC-EV-treated group compared with those in the PBS-treated group. Compared to the autophagy-inactive SLE-EV treatment group, the Rapa-SLE-EV treatment group showed significantly reduced plasma levels of BAFF, IL-4, IL-5, GM-CSF, and CXCL1; these effects were not observed in the FBS-treated groups. Treatment with Rapa-SLE-EV also significantly reduced the plasma levels of BAFF, IL-17A, IL-12p70, IL-4, IL-5, GM-CSF, and CXCL2. Meanwhile, Rapa-SLE-EV significantly increased plasma IL-10 levels compared with the other treatments (Fig. 4I).

These findings suggest that the autophagy activation enhances MSC-EV function in the SLE microenvironment and attenuates the plasma inflammatory environment milieu by reducing pro-inflammatory cytokine levels.

### Autophagy activation enhances the protective effect of MSC-EVs on renal function and pathological injury

**Fig. 5:**
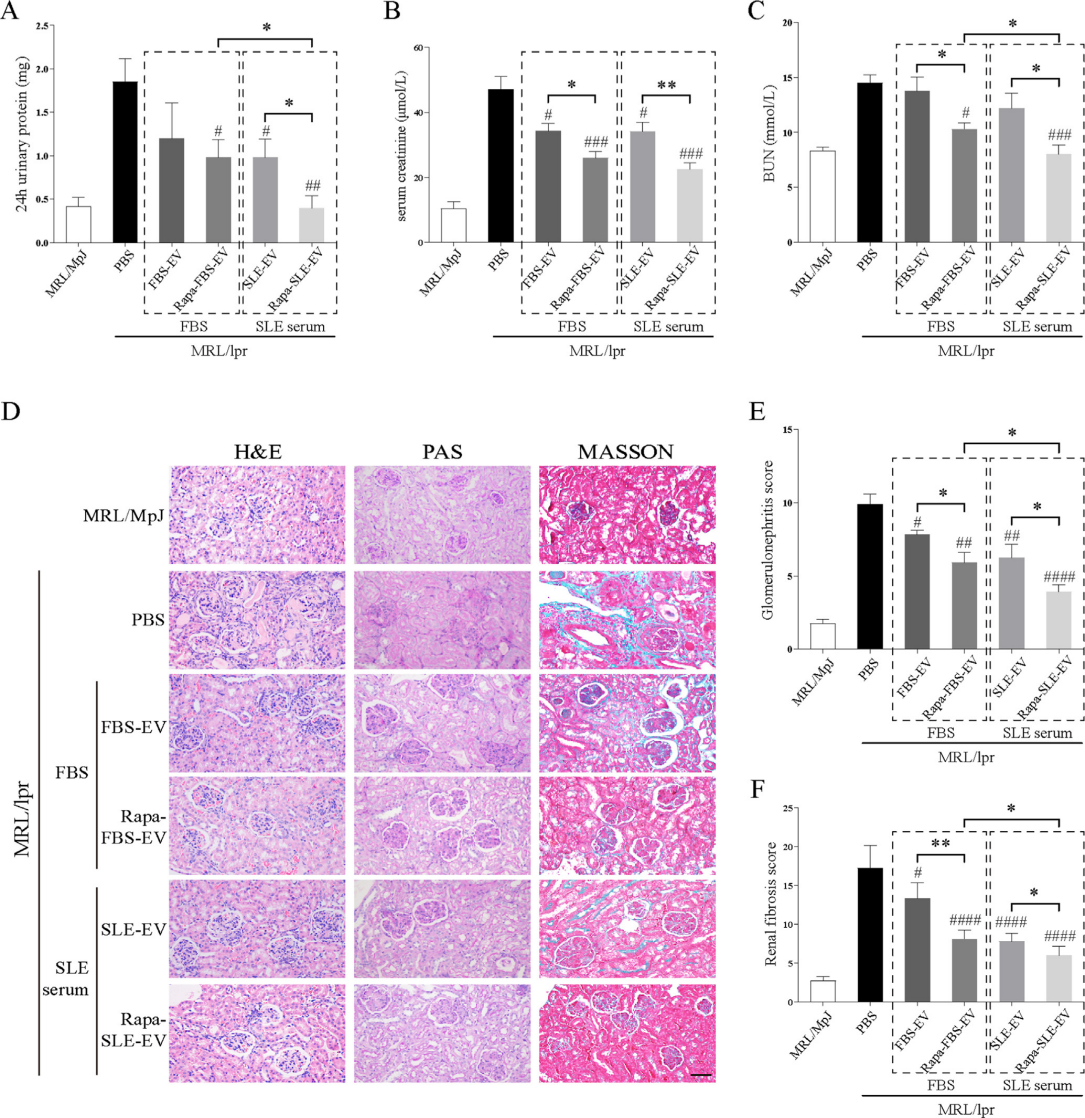
**Autophagy activation enhances the protective effect of MSC-EVs on renal function and pathological injury.** (A) Concentration of 24-h urinary protein. (B) Level of serum creatinine. (C) Level of BUN. (D) Representative histological images of renal hematoxylin and eosin, periodic acid-Schiff, and Masson's staining. Scale bars = 100  µm. (E) Quantitative analysis of the glomerulonephritis score. (F) Quantitative analysis of the renal fibrosis score. ^#^*P* < 0.05, ^##^*P* < 0.01, ^###^*P* < 0.001, ^####^*P* < 0.0001 *vs* the PBS group. *P < 0.05, **P < 0.01, ***P < 0.001, ****P < 0.0001 between groups. FBS-EVs: from MSCs cultured in FBS; Rapa-FBS-EVs: from MSCs cultured with rapamycin and FBS; SLE-EVs: from MSCs cultured with SLE serum; Rapa-SLE-EVs: from MSCs cultured with rapamycin and SLE serum.

Lupus is an autoimmune disease affecting multiple organs, and LN is one of the most common complications of SLE. Compared to MRL/MpJ mice, MRL/lpr mice showed a certain degree of impairment in renal function, including 24-h urinary protein, serum creatinine, and blood urea nitrogen (BUN). Autophagy-activated Rapa-FBS-EVs and Rapa-SLE-EVs significantly reduced serum creatinine and BUN levels in MRL/lpr mice. Rapa-SLE-EVs were more effective than Rapa-FBS-EVs in reducing the 24-h urinary protein and BUN levels (Fig. 5A–C).

Pathological staining further revealed the protective effect of MSC-EV treatment on renal pathology (Fig. 5D). Obvious glomerular inflammatory infiltration and renal fibrosis was detected in MRL/lpr mice. These effects were reduced following MSC-EV treatment, with and without autophagy activation. Moreover, Rapa-FBS-EVs and Rapa-SLE-EVs significantly reduced the glomerulonephritis score and renal fibrosis score. However, Rapa-SLE-EVs were more effective than Rapa-FBS-EVs (Fig. 5E and F).

### Autophagy activation enhances the inhibitory effect of MSC-EVs on glomerular immune complex deposition

**Fig. 6:**
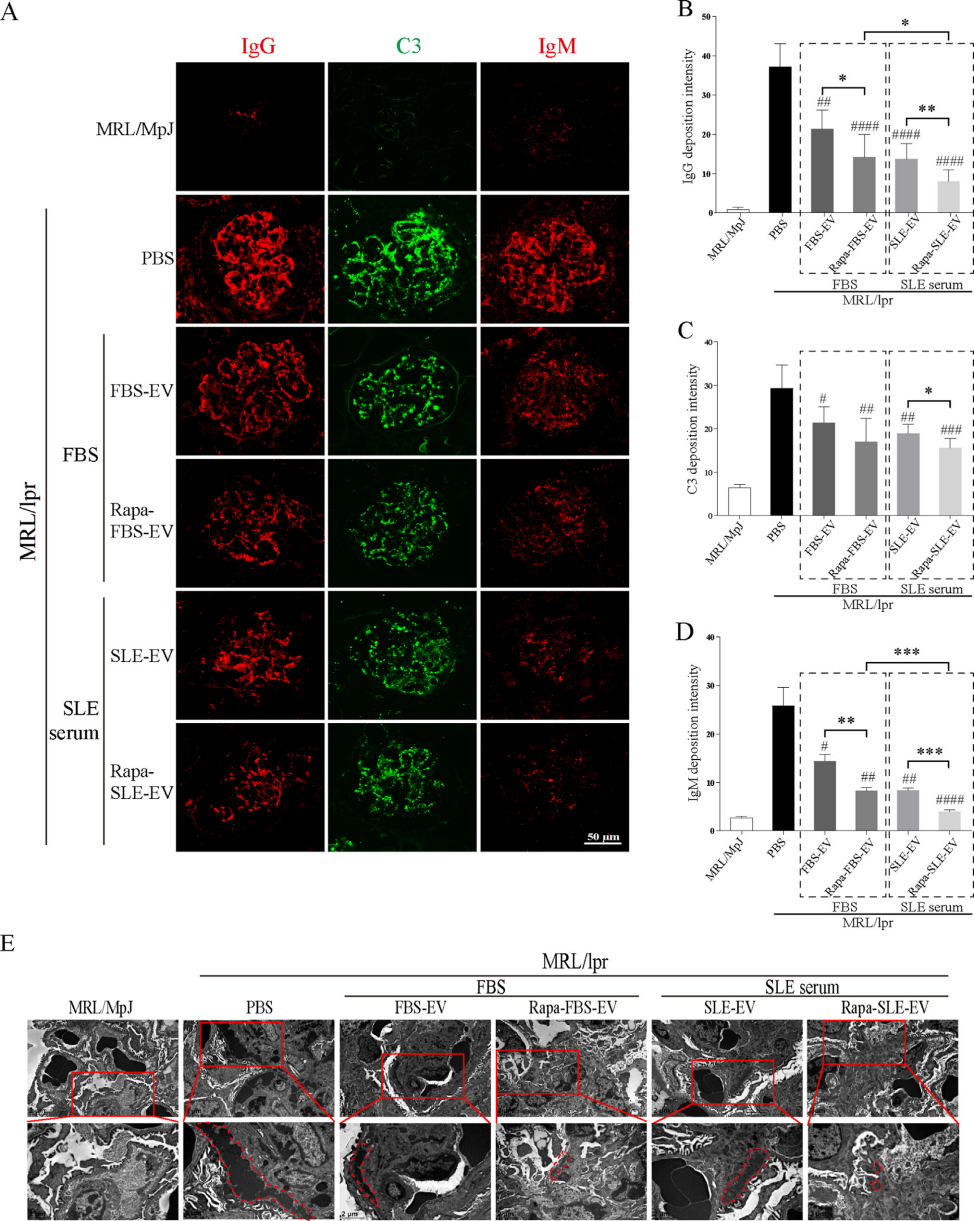
**Autophagy activation enhances the inhibitory effect of MSC-EVs on glomerular immune complex deposition.** (A) Representative immunofluorescence images of glomerular IgG, C3, and IgM. Scale bars = 50  µm. (B) Quantitative analysis of the glomerular deposition of IgG. (C) Quantitative analysis of the glomerular deposition of C3. (D) Quantitative analysis of the glomerular deposition of IgM. (E) Representative TEM images of electron-dense deposits. Red dashed line: electron-dense deposits. Scale bars = 2 µm. ^#^*P* < 0.05, ^##^*P* < 0.01, ^###^*P* < 0.001, ^####^*P* < 0.0001 *vs* the PBS group. *P < 0.05, **P < 0.01, ***P < 0.001, ****P < 0.0001 between groups. FBS-EVs: from MSCs cultured in FBS; Rapa-FBS-EVs: from MSCs cultured with rapamycin and FBS; SLE-EVs: from MSCs cultured with SLE serum; Rapa-SLE-EVs: from MSCs cultured with rapamycin and SLE serum. (For interpretation of the references to colour in this figure legend, the reader is referred to the web version of this article.)

Immunofluorescence results indicated that MSC-EV-treated mice had significantly reduced deposits of IgG, C3, and IgM in the glomeruli (Fig. 6A). Compared with FBS-EVs and Rapa-FBS-EVs, Rapa-FBS-EVs and Rapa-SLE-EVs significantly reduced the glomerular deposition of IgG and IgM (Fig. 6B and D). However, only Rapa-SLE-EVs exerted a significant effect on C3 deposition (Fig. 6C). Moreover, TEM results revealed extensive electron-dense deposits along the glomerular basement membrane in PBS-treated mice; these deposits were significantly reduced in MSC-EV-treated mice. However, the reduction in the SLE-EV, Rapa-FBS-EV, and Rapa-SLE-EV groups was more significantly than in the FBS-EV group (Fig. 6E). These results suggest that autophagy activation helps inhibit immune complex deposition by MSC-EV and that autophagy activation in SLE serum further enhances this inhibition.

### Autophagy activation enhances the inhibitory effect of MSC-EVs on B cells from SLE patients

**Fig. 7:**
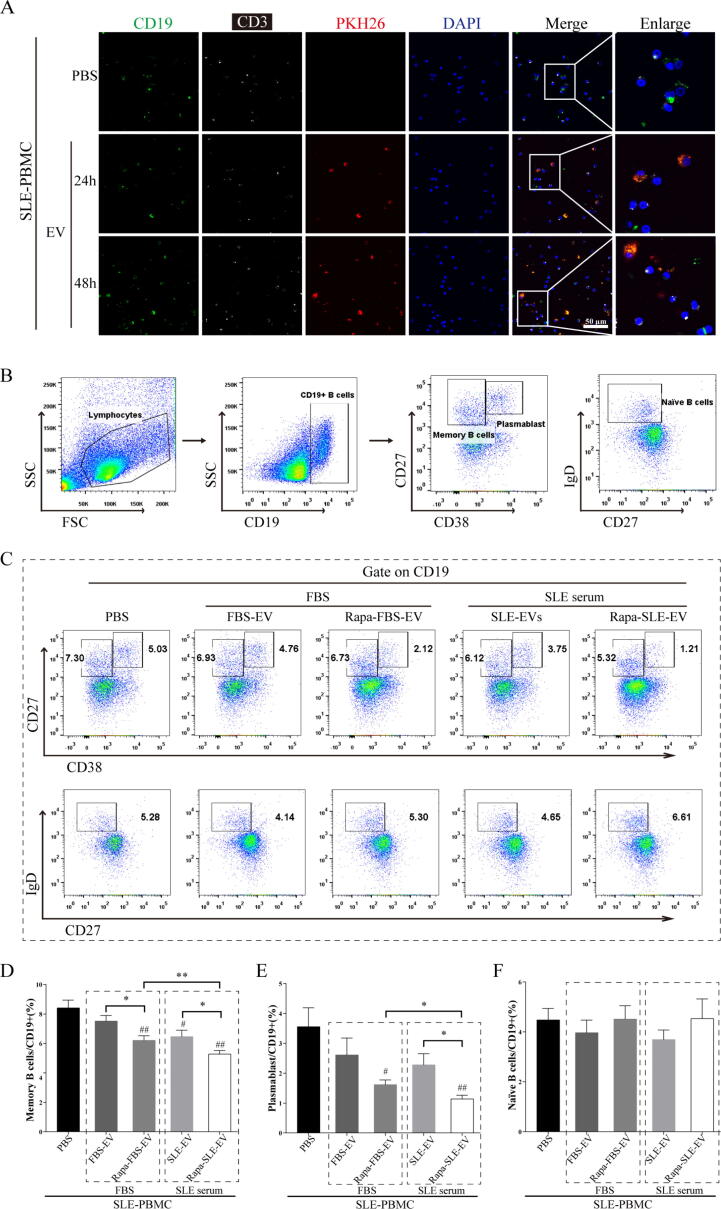
**Autophagy activation enhances the inhibitory effect of MSC-EVs on B cells of patients with SLE.** (A) Representative immunofluorescence images of MSC-EVs labeled with PKH-26 in SLE PBMCs. Scale bars = 50  µm. (B) Flow cytometry gating strategy. (C) Representative flow cytometry images of CD19^+^ CD27^+^ CD38^-^ Memory B cells, CD19^+^ CD27^hi^ CD38^hi^ Plasmablasts, and CD19^+^ IgD^+^ CD27^-^ naïve B cells. (D) Proportion of memory B cells in CD19^+^ B cells. (E) Proportion of plasmablasts in CD19^+^ B cells. (F) Proportion of naïve B cells in CD19^+^ B cells. Means ± SD of n = 5 patient samples. Scale bars = 50 µm. ^#^*P* < 0.05, ^##^*P* < 0.01 *vs* the PBS group. *P < 0.05, **P < 0.01 between groups. FBS-EVs: from MSCs cultured in FBS; Rapa-FBS-EVs: from MSCs cultured with rapamycin and FBS; SLE-EVs: from MSCs cultured with SLE serum; Rapa-SLE-EVs: from MSCs cultured with rapamycin and SLE serum.

B cells are central to the pathogenesis of SLE through antigen presentation, cytokine secretion, and the production of autoantibodies [27], [28], [29]. After co-cultured labeled MSC-EVs with PBMCs from patients with active-SLE, CD19^+^ B cells took up PKH26-labeled MSC-EVs earlier and more often than CD3^+^ T cells (Fig. 7A). Subsequently, we compared the inhibitory effects of different MSC-EV on B cells from SLE patients (Fig. 7B). The MSC-EV-treated groups had a significantly lower proportion of memory B cells (CD19^+^CD27^+^CD38^–^) than the PBS group (PBS: 8.42 ± 1.17 %, FBS-EV: 7.53 ± 0.85 %, Rapa-FBS-EV: 6.22 ± 0.69 %, SLE-EV: 6.47 ± 0.98 %, Rapa-SLE-EV: 5.28 ± 0.53 %). Furthermore, memory B cells were significantly reduced in the Rapa-FBS-EV and Rapa-SLE-EV groups compared with the FBS-EV and SLE-EV groups, respectively (Rapa-FBS-EV *vs.* FBS-EV, *P* < 0.05; Rapa-SLE-EV *vs.* SLE-EV, *P* < 0.05). Fewer memory B cells were also observed in the Rapa-SLE-EV group compared with the Rapa-FBS-EV group (Rapa-SLE-EV *vs.* Rapa-FBS-EV, *P* < 0.01; Fig. 7C and D).

The MSC-EV-treated groups had a significantly lower proportion of plasmablasts (CD19^+^CD27^hi^CD38^hi^) than the PBS group (PBS: 3.56 ± 1.42 %, FBS-EV: 2.61 ± 1.27 %, Rapa-FBS-EV: 1.62 ± 0.35 %, SLE-EV: 2.28 ± 0.83 %, Rapa-SLE-EV: 1.14 ± 0.27 %). Additionally, plasmablasts were significantly reduced in the Rapa-SLE-EV group (Rapa-SLE-EV *vs.* SLE-EV, *P* < 0.05; Rapa-SLE-EV *vs.* Rapa-FBS-EV, *P* < 0.05; Fig. 7C and E). However, changes were not observed in the proportion of naïve B cells (CD19^+^IgD^+^CD27^-^; Fig. 7C and F).

**Fig. 8:**
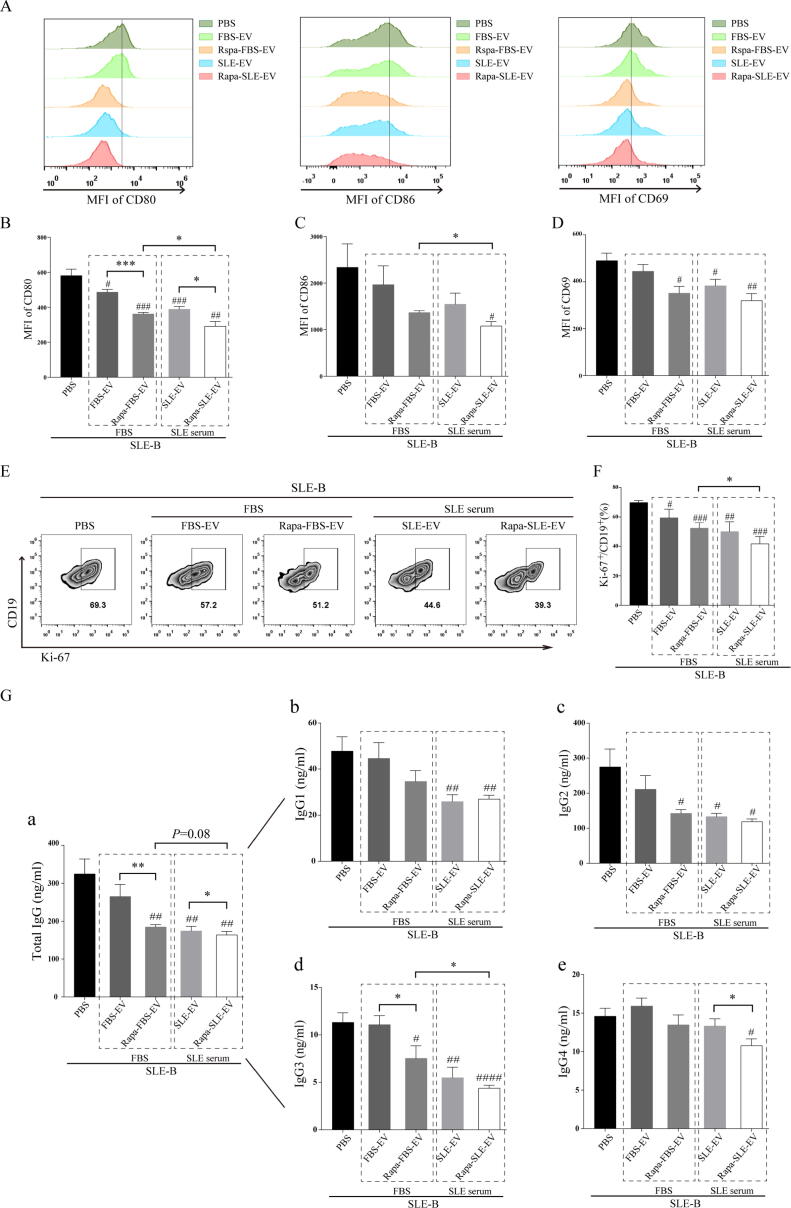
**Autophagy activation enhances the inhibitory effect of MSC-EVs on B cells from patients with SLE.** (A) Representative flow cytometry images of the mean fluorescence intensity (MFI) for CD80, CD86, and CD69. (B–D) Statistics of the MFI for CD80, CD86, and CD69. (E) Representative flow cytometry images of Ki-67^+^ B cells in CD19^+^ B cells. (F) Proportion of Ki-67^+^ B cells in CD19^+^ B cells. (G) Effect of MSC-EV treatment on SLE-B cell in antibody secretion. Concentration of (a) total IgG, (b) IgG1, (c) IgG2, (d) IgG3, and (e) IgG4. Means ± SD of n = 5 patient samples. ^#^*P* < 0.05, ^##^*P* < 0.01, ^###^*P* < 0.001 *vs* the PBS group. *P < 0.05 between groups. FBS-EVs: from MSCs cultured in FBS; Rapa-FBS-EVs: from MSCs cultured with rapamycin and FBS; SLE-EVs: from MSCs cultured with SLE serum; Rapa-SLE-EVs: from MSCs cultured with rapamycin and SLE serum.

To investigate the inhibitory effect of MSC-EVs on B-cell activation, B cells were isolated from the peripheral blood of patients with active SLE. All groups of MSC-EVs inhibited B-cell activation *in vitro*. Compared with the other groups, Rapa-SLE-EV treatment inhibited B-cell activation most significantly, as evidenced by the expression of costimulatory molecules CD80 and CD86 (Fig. 8A–D). Moreover, MSC-EVs inhibited B-cell proliferation *in vitro*, with Rapa-SLE-EV treatment exhibiting the strongest inhibittory (Fig. 8E and F). A prominent feature of SLE involves the anomalous release of antibodies by B cells following activation, with a predominant contribution from IgG antibodies in the atypical secretion process. The administration of Rapa-SLE-EVs also markedly reduced the secretion of IgG antibodies by SLE-B cells, with a particular suppression of total IgG antibodies (Fig. 8G).

These findings suggest that autophagy activation enhances the immunosuppressive function of MSC-EVs on SLE-B cells. This further emphasizes that autophagy activation in the SLE microenvironment may promote MSC-EV function.

### The immunosuppressive protein IDO1 is differentially enrich in autophagy-activated MSC-EVs

**Fig. 9:**
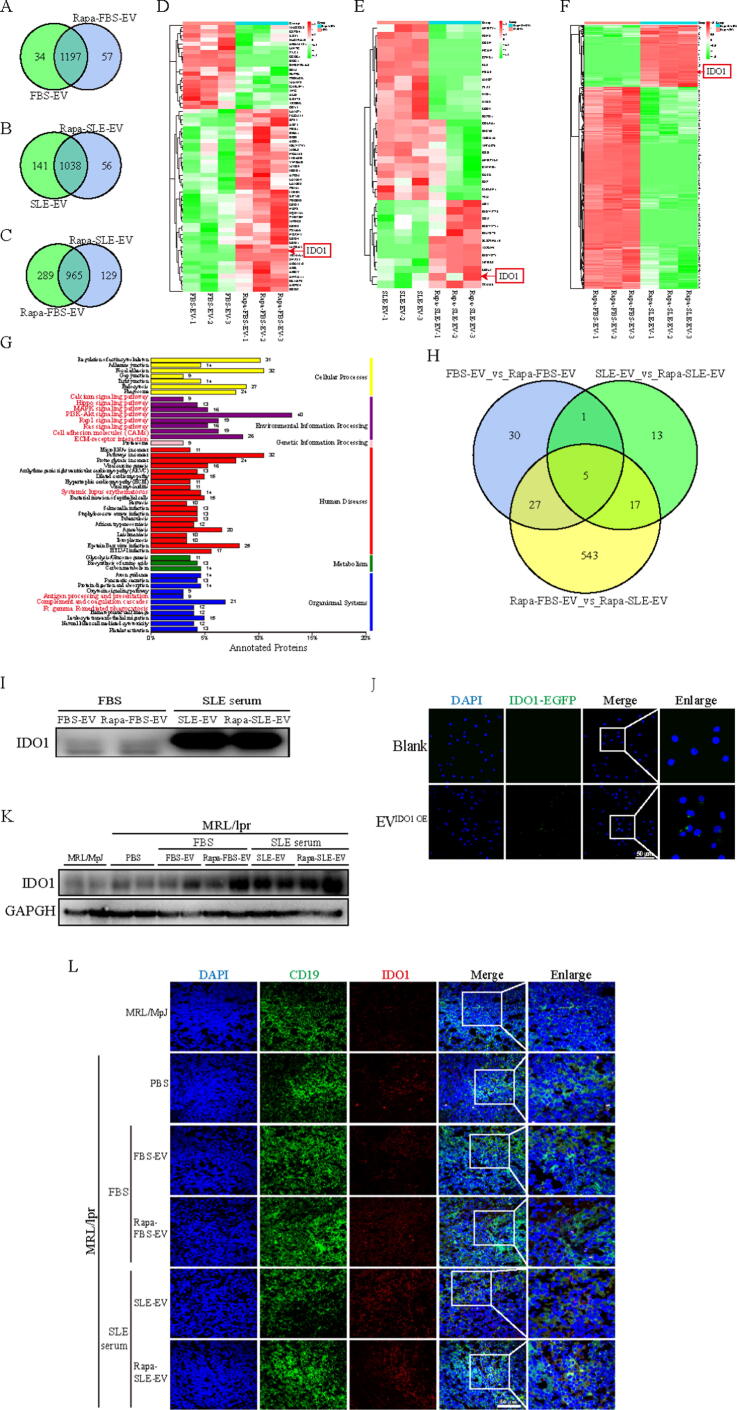
**The immunosuppressive protein IDO1 is differentially enriched in autophagy-activated MSC-EVs.** (A–C) Venn diagrams comparing the protein composition of FBS-EV to Rapa-FBS-EV, SLE-EV to Rapa-SLE-EV, and Rapa-FBS-EV to Rapa-SLE-EV, respectively. (D–F) Heatmaps of the differential protein expression between FBS-EV and Rapa-FBS-EV, SLE-EV and Rapa-SLE-EV, and Rapa-FBS-EV and Rapa-SLE-EV, respectively. (G) Number of differentially expressed proteins in each KEGG pathway. (H) Venn plot of the common differential proteins between pairs. (I) Differential expression of IDO1 in different MSC-EVs. (J) Effect of Rapa-SLE-EVs on the entry of IDO1-EGFPs into SLE-B cells. (K) IDO1 expression in the spleen of mice treated with different MSC-EVs. (L) IDO1 expression in CD19^+^ B cells from mouse spleens treated with different MSC-EVs. Scale bars = 50 µm. FBS-EVs: from MSCs cultured in FBS; Rapa-FBS-EVs: from MSCs cultured with rapamycin and FBS; SLE-EVs: from MSCs cultured with SLE serum; Rapa-SLE-EVs: from MSCs cultured with rapamycin and SLE serum.

Proteomic analysis revealed that the four types of exosomes subjected to different treatments had unique proteins profiles (Fig. 9A–C). In particular, 965 proteins were shared between the Rapa-FBS-EVs and Rapa-SLE-EVs, while 289 and 129 proteins were unique to Rapa-FBS-EVs and Rapa-SLE-EVs, respectively (Fig. 9C). Compared with FBS-EVs, Rapa-FBS-EVs had 64 differentially expressed proteins, 44 were upregulated, and 20 were downregulated. Compared with SLE-EVs, Rapa-SLE-EVs has 36 differentially expressed proteins, including 12 upregulated and 14 downregulated. Compared with Rapa-FBS-EVs, Rapa-SLE-EVs had 592 differentially expressed proteins, including 139 upregulated and 453 downregulated (Fig. 9D–F).

The proteins differentially expressed in Rapa-SLE-EVs were classified according to the KEGG pathways. Differential proteins were enriched in inflammation-related pathways, including phosphatidylinositol 3-kinase (PI3K)/protein kinase B (AKT), mitogen-activated protein kinase (MAPK), rat sarcoma virus (RAS), and Hippo signaling pathways. Pathways related to immune regulation, such as antigen processing and presentation, complex and coagulation cascade, and Fc gamma R-mediated phagocytosis, were also enriched. Additionally, 14 differentially expressed proteins were related to SLE in the human disease (Fig. 9G). Proteomic analysis identified that Rapa-SLE-EVs are rich in various proteins involved in immune regulation and anti-inflammatory properties, including Indoleamine 2,3-dioxygenase 1 (IDO1), cathepsin G (CTSG), CD276, Milk Fat Globule EGF And Factor V/VIII Domain Containing (MFGE8), and Complex-related components (Fig. 9H). Of particular importance is IDO1, which has been widely proven to be a key participant in MSC-mediated immune regulation [30], [31]. It has also been proven to be enriched in MSC-EV [8], [32].

Notably, IDO1 was upregulated in Rapa-FBS-EVs and Rapa-SLE-EVs; however, it was more enriched in Rapa-SLE-EVs. This was confirmed by western bloting (Fig. 9I). To determine whether IDO1 in Rapa-SLE-EVs can enter target cells, an IDO1-EGFP-based adenovirus vector was generated. Immunofluorescence staining confirmed that IDO1-EGFP entered B cells through EVs and exerted its function (Fig. 9J). In addition, a similar increase in IDO1 expression was observed in the splenic tissue of mice treated with Rapa-SLE-EVs through immunoblotting and immunofluorescence staining (Fig. 9K and L).

**Fig. 10:**
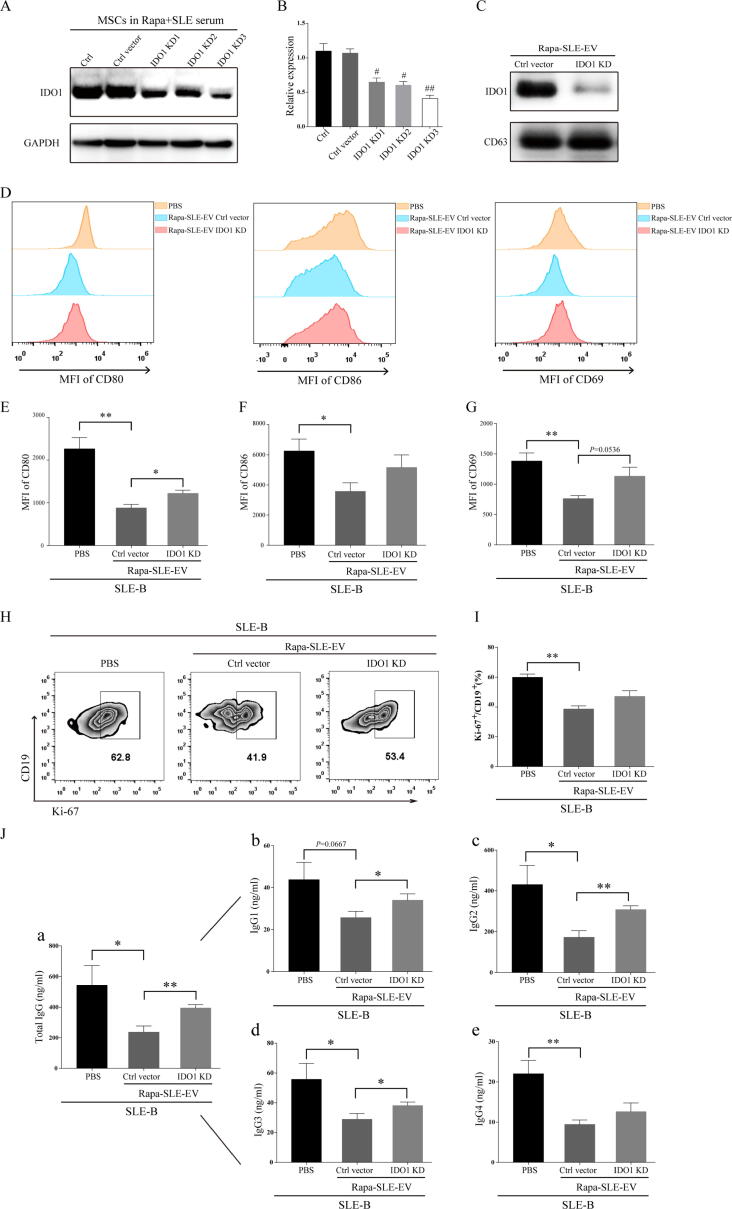
**Downregulating IDO1 expression impacts the inhibitory efficacy of Rapa-SLE-EVs on SLE-B cells.** (A) Representative western blot of IDO1 in MSCs. (B) Statistical analysis of IDO1 expression. (C) Representative western blot of IDO1 in Rapa-SLE-EVs. (D) Representative flow cytometry images of MFI of CD80, CD86, and CD69. (E–G) Statistics of CD80, CD86, and CD69 MFIs. (H) Representative flow cytometry images of Ki-67^+^ B cells in CD19^+^ B cells. (I) Proportion of Ki-67^+^ B cells in CD19^+^ B cells. (J) Effect of IDO1 knockdown on the inhibitory effect of Rapa-SLE-EVs on antibody secretion in SLE-B cells. Concentration of (a) total IgG, (b) IgG1, (c) IgG2, (d) IgG3, and (e) IgG4. Means ± SD of n = 5 patient samples. *P < 0.05, **P < 0.01. Rapa-SLE-EVs: from MSCs cultured with rapamycin and SLE serum.

To investigate whether IDO1 carried in Rapa-SLE-EVs inhibited SLE-B cells, it expression was knocked down in Rapa-SLE-EVs (Fig. 10A–C). Purified SLE-B cells were treated with Rapa-SLE-EVs transfected with a control vector or IDO1-knockdown vector. Compared to the control vector group, Rapa-SLE-EVs with IDO1 knockdown partially reversed the inhibitory effects of Rapa-SLE-EVs on SLE-B cells activation (Fig. 10D–G), proliferation (Fig. 10H and I), and IgG antibody secretion (Fig. 10J). These data suggested that IDO1 downregulation influenced the inhibitory effects of Rapa-SLE-EVs on SLE-B cells.

## Discussion

In this study, we used autophagy activation combined with SLE serum-treated MSC-EVs to treat SLE. Autophagy activation combined with SLE serum enhanced the ability of MSC-EVs to reduce autoantibodies and improve renal function in a spontaneous MRL/lpr mouse model. In addition, *in vitro*, MSC-EVs inhibited the differentiation, activation, and proliferation of B cells derived from patients with SLE. Mechanistically, Rapa-SLE-EVs exhibited IDO1 expression, which was taken up by SLE-B cells, contributing to their functional inhibition.

Increasing evidence suggests that the immunomodulatory therapeutic effects of MSC administration are primarily due to the paracrine mechanisms mediated by MSC-EVs [33], [34], [35]. EVs have many advantages in tissue engineering and drug delivery; furthermore, they do not induce immune rejection, tumorigenicity, or vascular obstruction [36], [37]. In addition to promoting tissue repair, MSC-EVs exert broad immunosuppressive effects by inhibiting the functions of T and B cells, natural killer cells, dendritic cells, monocytes, and macrophages [38], [39]. Notably, MSC-EVs exert therapeutic effects on graft-versus-host disease [40] and chronic kidney disease [41]. Various preclinical models of immune diseases have shown that MSC-EVs are therapeutically effective. MSC-EVs also improve lupus functions in various experimental models [42], [43], [44], [45]. However, evidence regarding the administration of MSC-EVs in SLE animal models and clinical trials remains limited. Therefore, further research is necessary to determine whether MSC-EVs can be effectively translated into clinical treatments for SLE patients.

Moreover, EV biogenesis and cargo selection are regulated by environmental specificity; different conditions for cultivating parental cells may trigger unique functions in homologous EVs [46]. EVs derived from immune cells can target and localize to pathological or inflammatory cells by interacting with distinct surface protein signatures [47]. Therefore, enhancing the function of MSC-EVs through *in vitro* pretreatment or modification is an effective method for slowing the SLE progression and improving LN. Simulating the inflammatory scenario in which MSC transplantation treats diseases can more realistically explore the therapeutic potential of MSC-EVs. It also contributes to the acquiring of EVs with higher immunomodulatory functions in different pathophysiological settings.

The paracrine effect of MSCs is directly related to the pathological conditions of the disease microenvironment or *in vitro* culture conditions. Currently, traditional culture conditions are commonly used to cultivate MSCs and isolate EVs for disease treatment. SLE is among the most complex autoimmune diseases, making it difficult to predict which serum cytokines impact the immunosuppressive potential of MSCs. Clinically, the SLE microenvironment significantly affects the composition and biological function of EVs during intravenous MSC infusion. Therefore, we used serum obtained from patients with SLEDAI > 6 to culture MSCs, simulating the effect of the SLE microenvironment on MSC paracrine secretion. The SLE serum triggered an autophagic response in MSCs, leading us to speculate that the influence of the SLE microenvironment on MSCs or EVs might be related to an increase in autophagy levels in MSCs. This was supported by an increase in autophagic vacuoles and LC3-II/I ratio in MSCs following SLE serum treatment. However, an increase in the autophagy substrate P62 suggested impaired autophagy, potentially due to the complex components of SLE serum obstructing autophagic flux in MSCs. Therefore, rapamycin was used to further activate autophagy, reversing the increase in P62 protein expression and further increasing the LC3-II/I ratio.

Considering the critical role of autophagy in the therapeutic effects of MSCs [48], we speculate that enhanced autophagy in an SLE environment improves MSC-EV function. According to our data, the therapeutic effect of Rapa-SLE-EVs was superior to that of Rapa-FBS-EVs, especially regarding inhibiting lymphatic hyperplasia and protecting kidney function. Rapa-SLE-EVs significantly reduced pathogenic dsDNA IgG autoantibodies, inhibited lymphadenopathy and splenomegaly, inhibited B-cell differentiation, reduced serum pro-inflammatory factors, and alleviated kidney pathological injury and immune complex deposition. *In vitro,* co-cultures with B cells revealed the immunosuppressive capacity of Rapa-SLE-EVs.

MSCs exist in a state of 'arrested' autophagy, while rapamycin can stimulate autophagy activation and inhibit differentiation [49], [50], [51], [52], [53]. This is, rapamycin reverses the senescent phenotype and improves the immuno-regulation of MSCs from MRL/lpr mice by inhibiting the mTOR signaling pathway [54]. In the current study, MSCs were in a state of arrested autophagy when cultured with in FBS, whereas the autophagic response was triggered in the SLE serum culture. MSCs are affected by multiple complex components of the SLE microenvironment that activate autophagy. Autophagy and EVs share molecular mechanisms and engage in crosstalk [55]. The autophagy protein LC3 coupling pathway is necessary for EV loading and RNA binding protein secretion [56]. In addition to its traditional role in maintaining protein, lipid, and organelle homeostasis, autophagy can also affect RNA homeostasis [57]. Moreover, the MSC-EVs from the four study groups exhibited distinct surface proteins and characteristics. This suggested that autophagy may be involved in the packaging and synthesis of MSC-EVs, affecting their immunosuppressive function.

Previous proteomic analyses of EVs have identified multiple proteins involved in immune responses or possessing anti-inflammatory properties [58], [59]. To further investigate the potential role of autophagy activation on SLE serum-treated MSC-EV proteins in the clinical treatment of SLE, we performed proteomic analyses of different MSC-EVs. Our data indicates that autophagy activation and SLE serum microenvironment significantly regulate the protein composition of EVs. KEGG pathway enrichment analysis showed that the differentially expressed proteins within EVs derived from MSCs treated with rapamycin and SLE serum were associated with immunosuppression, such as IDO1, CTSG, CD276, and complex-related components. We observed a significant increase in IDO1 expression in Rapa-SLE-EVs as well as in the splenic tissue of mice treated with Rapa-SLE-EVs. Meanwhile, knocking down IDO1 in Rapa-SLE-EVs partially reversed the inhibitory effects on SLE-B cells, especially significantly affecting the suppression of antibody secretion. This suggests that combining of autophagy activation with SLE serum treatment enhances the immune regulatory function of MSC-EVs, which may be partially driven by upregulated IDO1 expression. Indeed, IDO1 suppresses inflammatory B cell responses, which may help control autoimmune reactions and excessive inflammation [60]. Research on IDO1 and IDO2 knockout in mouse B cells has provided valuable insights into their distinct roles in B cell-mediated immune responses. It has been demonstrated that IDO2 knockout inhibits the production of autoantibodies and alleviates symptoms in arthritis models, suggesting that IDO2 acts as a pro-inflammatory mediator directly within B cells, while IDO1 has markedly different functions [61]. IDO1 is associated with immune regulation, as it can deplete local tryptophan and produce immunoregulatory tryptophan catabolites. In particular, the key tryptophan metabolite kynurenine (KYN) suppresses immune cell function [62]. KYN also activates the aryl hydrocarbon receptor (AhR), inducing immunosuppression [63], [64], [65]. Amino acid-sensing enzymes, such as GCN2 and mTOR may also contribute to IDO1-mediated tryptophan depletion, cell cycle arrest, and promotion of CD4^+^ T cell differentiation into regulatory T cells [66], [67], [68]. Microbiota-derived metabolites suppress arthritis by amplifying AhR activation in regulatory B Cells [69]. Increased AhR activity is associated with autoimmune disease pathology and preclinical disease models, with AhR agonists suppressing inflammation in models of inflammatory bowel disease, SLE, and rheumatoid arthritis [70], [71]. We hypothesize that the heightened expression of IDO1 in Rapa-SLE-EVs potentially mediates immunosuppressive effects on SLE-B cells through analogous pathways. Future research should explore and validate the molecular mechanisms involved in the interaction between SLE-B cell and IDO1.

This study has certain limitations regarding the experimental design and unaccounted potential variables. To provide further evidence regarding the role and related mechanisms of autophagy activation in enhancing the therapeutic effect of SLE serum-treated MSC-EVs on SLE, future research should optimize strategies and treatment timing, especially for patients with active SLE. Attention should be paid to the potential benefits of combination therapy in alleviating autoimmune reactions and delaying the progression of SLE. It is also important to further characterize how dosage and injection frequency influence the outcomes, particularly since increasing the MSC dose has yielded mixed findings.

## Conclusions

This study confirms that the immunomodulatory function of Rapa-SLE-EVs is enhanced through autophagy activation and exposure to SLE serum. This effect is partially attributed to increased expression of the anti-inflammatory protein IDO1 in B cells. These findings provide new research directions for the improved application of MSC-EVs in treating immune-related diseases, including SLE.

## CRediT authorship contribution statement

**Shuzhen Liao:** Investigation, Conceptualization, Data curation, Methodology, Validation, Visualization, Writing - original draft. **Fengbiao Guo:** Conceptualization, Methodology, Writing - original draft, Funding acquisition. **Zengzhi Xiao:** Investigation, Conceptualization, Validation, Writing - original draft. **Haiyan Xiao:** Investigation, Resources, Formal analysis. **Quan-ren Pan:** Investigation, Resources, Validation. **Yugan Guo:** Resources, Visualization, Software. **Jiaxuan Chen:** Formal analysis, Visualization. **Xi Wang:** Formal analysis, Visualization. **Shuting Wang:** Validation. **Haimin Huang:** Visualization. **Lawei Yang:** Formal analysis. **Hua-feng Liu:** Methodology, Formal analysis, Visualization, Funding acquisition, Project administration. **Qingjun Pan:** Conceptualization, Methodology, Project administration, Supervision, Funding acquisition, Writing - review & editing.

## Compliance with ethics requirements

**Ethics approval and consent to participate**

All animal experiments were approved by the institutional Laboratory Animal Ethical Committee (LAEC; Approval no. GDY2103031). The Guangdong Medical University LAEC reviewed animal welfare during the application process of animal experiments and animal experiments. All patients recruited for this study followed a protocol approved by the Human Ethics Committee of the Affiliated Hospital of Guangdong Medical University (Approval no. YJYS2021069) and obtained informed written consent.

## *Declaration of competing interest*

The authors declare that they have no known competing financial interests or personal relationships that could have appeared to influence the work reported in this paper.

## Data Availability

Data will be made available on request.
